# P-48. Uptake of pneumococcal vaccination after invasive pneumococcal diseases in two centers in northern Italy

**DOI:** 10.1093/ofid/ofae631.255

**Published:** 2025-01-29

**Authors:** Giacomo Ponta, Angelo Roberto Raccagni, Silvia Carletti, Carola Mauri, Valentina Morena, Nicole Gemignani, Silvia Pontiggia, Marco Ripa, Antonella Castagna, Stefania Piconi

**Affiliations:** San Raffaele University Hospital, Milan, Lombardia, Italy; San Raffaele University Hospital, Milan, Lombardia, Italy; San Raffaele University Hospital, Milan, Lombardia, Italy; ASST Lecco A. Manzoni Hospital, Lecco, Lombardia, Italy; ASST Lecco A. Manzoni Hospital, Lecco, Lombardia, Italy; ASST Lecco A. Manzoni Hospital, Lecco, Lombardia, Italy; ASST Lecco A. Manzoni Hospital, Lecco, Lombardia, Italy; San Raffaele University Hospital, Milan, Lombardia, Italy; IRCCS San Raffaele Hospital and Vita-Salute San Raffaele University, Milano, Lombardia, Italy; ASST Lecco A. Manzoni Hospital, Lecco, Lombardia, Italy

## Abstract

**Background:**

Aim of this study is to evaluate factors associated with uptake of pneumococcal vaccination after invasive pneumococcal disease (IPD). Since pneumococcal infection doesn’t protect from future ones, vaccination is recommended in patients who recover from IPD.

Table 1

**Figure:**
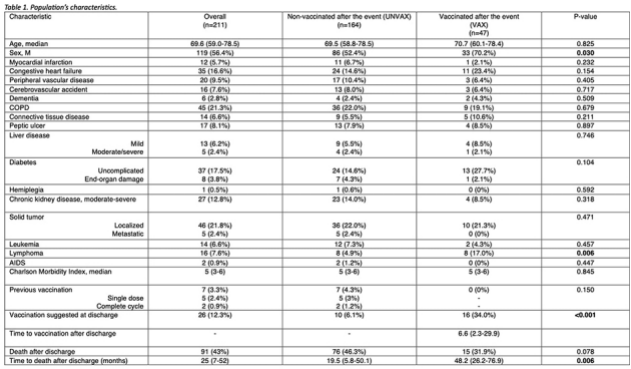

Population's characteristics.

**Methods:**

Retrospective, bicenter cohort study of patients hospitalized with invasive pneumococcal disease at IRCCS San Raffaele Hospital (Milan, Italy) and ASST Manzoni Hospital (Lecco, Italy) between January 1^st^ 2015 and December 31^st^ 2019. Patients older than 18 years with positive blood or cerebrospinal fluid cultures for *Streptococcus pneumoniae* were included. Patients who died before discharge and whose vaccinal record and discharge documentations were not available were excluded. All vaccination data were retrieved through Italian national vaccination portal (SIAVR). Follow-up data were updated until March 31^st^, 2024.

The characteristics of patients who were vaccinated (VAX) and were not vaccinated (UNVAX) after the event were compared using the chi-square test for categorical variables and the Mann-Whitney U-test for continuous variables.

Table 2

**Figure:**
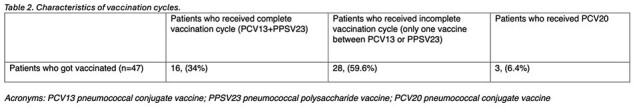

Characteristics of vaccination cycles.

**Results:**

The characteristics of the 211 included patients are described in *table 1,* completed vaccination cycles in *table 2*. Only 26 patients (12.3%) received an indication at discharge to get vaccinated for *S. pneumoniae*, and 47 (22.2%) patients were vaccinated during the observation period, after a median of 6.6 (2.3-29.9) months after the event. There was no difference in age between the two groups (70.7 [60.1-78.4] vs 69.5 [58.8-78.5], p-value=0.82), while a greater proportion of VAX patients was male (33 [70.2%] vs 86 [52.4%], p-value=0.03). Charlson comorbidity index was similar between the two groups (5 [3-6] vs 5 [3-6], p-value=0.845), but a significant higher percentage of VAX patients had lymphoma (8 [17%] vs 8 [4.9%], p-value=0.006). Patients who received an indication to vaccinate were significantly more likely to receive pneumococcal vaccination (16 [34%] vs 10 [6.1%], p-value< 0.001).

**Conclusion:**

Our cohort revealed low pneumococcal vaccination uptake, with inadequate frequency of vaccination indication at discharge. Reinforcing prevention culture among patients and physicians is imperative.

**Disclosures:**

**Antonella Castagna, MD**, Bristol-Myers Squibb: Advisor/Consultant|Gilead Sciences, Inc.: Advisor/Consultant|Gilead Sciences, Inc.: Grant/Research Support|Gilead Sciences, Inc.: Honoraria|Janssen: Advisor/Consultant|Janssen: Grant/Research Support|Janssen: Honoraria|Merck Sharp & Dohme: Advisor/Consultant|Merck Sharp & Dohme: Grant/Research Support|Merck Sharp & Dohme: Honoraria|ViiV Healthcare: Advisor/Consultant|ViiV Healthcare: Grant/Research Support|ViiV Healthcare: Honoraria

